# Systematic Review of Effectiveness of Chitosan as a Biofunctionalizer of Titanium Implants

**DOI:** 10.3390/biology10020102

**Published:** 2021-02-01

**Authors:** Nansi López-Valverde, Antonio López-Valverde, Juan Manuel Ramírez

**Affiliations:** 1 Department of Surgery, Instituto de Investigación Biomédica de Salamanca (IBSAL), University of Salamanca, 37007 Salamanca, Spain; nlovalher@usal.es; 2 Department of Morphological Sciences, University of Cordoba, Avenida Menéndez Pidal s/n, 14071 Cordoba, Spain; jmramirez@uco.es

**Keywords:** titanium implant, chitosan, coating surface, functionalization

## Abstract

**Simple Summary:**

The low bioactivity of titanium limits its applications. The biofunctionalization of its surfaces with certain polymers could improve and accelerate the osseointegration process. Chitosan is a natural polysaccharide derived from chitin, which has been proposed in biomedical engineering. This systematic review evaluated in vivo studies with chitosan-coated titanium implants compared with non-functionalized implants.

**Abstract:**

Chitosan is a natural polysaccharide extracted from the shells of crustaceans that has been proposed as a scaffold in tissue engineering. Certain studies have proven a greater osseointegration of titanium surfaces that are functionalized with chitosan. The MEDLINE, CENTRAL, PubMed, and Web of Science databases were electronically searched for in vivo studies. Seven studies met the inclusion criteria. Animal models, implant site, chitosan incorporation methods, and methods of analysis were emphasized. The selected studies were individually discussed regarding the coatings, osseointegration potential, and suitability of the experimental models used, analyzing their limitations. We concluded that chitosan-biofunctionalized titanium surfaces have greater osseointegration capacity that uncoated control titanium alloys.

## 1. Introduction

The biofunctionalization of titanium (Ti) implants aimed at faster osseointegration has led researchers to develop different surfaces that can provide high osteogenic capacity [1]. Despite the osseointegration capacity of sandblasted, granulated, etched (SLA) surfaces, compared to machined surfaces, they require periods of 3 to 6 months to achieve adequate osseointegration [2]. Nevertheless, biofunctionalization using certain peptides, growth factors, nucleotides, or extracellular matrix proteins could lead to faster and more predictable osseointegration [3,4,5,6].

Chitosan (CS) is a natural polysaccharide derived from the partial deacetylation of chitin, a structural element found on the exoskeleton of crustaceans, insects, and on the cell walls of fungi, being the second most abundant natural polysaccharide after cellulose [7].

Its interesting qualities as a biodegradable, non-toxic, biocompatible, and immunotoxicity-free material, alongside its anticancer, antioxidant, and antimicrobial properties, allow it to be used for wound healing, as a drug carrier, in the management of obesity, or as a scaffold in tissue engineering [7,8,9,10,11].

The raw material used for its production is chitin, which conventionally demineralized, deproteinized, decolored, and finally highly purified, can be used for medical or pharmaceutical purposes. The conversion of chitin to CS is carried out through enzymatic or chemical deacetylation, the latter being the most common method of commercial preparation [12,13,14,15].

The poor bioactivity and deficient antibacterial properties of Ti surfaces can lead to failure and postoperative infections, limiting its applications [16,17,18], which is why it is necessary to modify Ti surfaces to improve their bioactivity.

There are currently different improvement methods, such as bioactive coatings and surface patterns (microstructures, nanostructures, micro-nanostructures); because of these well-proven benefits, such as the case with bioactive coatings, the use of Ti coatings has become one of the dominant approaches in the biomedical field to improve the osseointegration of dental implants [19].

The aim of our study was to conduct a systematic review of the scientific literature on in vivo studies related to the effectiveness of CS for the biofunctionalization of Ti surfaces aimed at improving osseointegration.

## 2. Materials and Methods

### 2.1. Protocol

The studies were selected according to the Preferred Reporting Items for Systematic Review and Meta-Analysis (PRISMA) guidelines for systematic reviews [20], formulating a specific question based on the PICO (Participants, Intervention, Control, Outcome) framework:

(P) Participants: Subjects received endosseous implantation;
(I) Intervention: Implants with chitosan incorporation;
(C) Control: Implants without chitosan incorporation;
(O) Outcome: Bone formation around the implant body.

The research question was: “Does the use of chitosan in titanium dental implant surfaces influence osseointegration?”.

### 2.2. Data Sources and Search Strategy

The MEDLINE, CENTRAL, PubMed, and Web of Science electronic databases were searched for findings published in the last 10 years until December 2020. The MeSH terms (Medical Subject Headings) used in MEDLINE, CENTRAL, and Pudmed data bases were: “titanium” [MeSH Terms], “implant” [MeSH Terms], “chitosan” [MeSH Terms], “coated materials, biocompatible” [MeSH Terms], “animals” [MeSH Terms]; the Boolean operator AND was used to refine the search. In Web of Science, the search terms were: “titanium implants”, “chitosan functionalized surface”, “chitosan coating surfaces”, “in vivo”; the Boolean operators AND, OR were used to refine the search (Table S1).

### 2.3. Inclusion and Exclusion Criteria

The inclusion criteria for the study selection were:

1. In vivo studies;
2. Studies where at least one layer of CS was used to coat the Ti;
3. Studies where bone growth or the formation of a biological seal around the Ti implant surface coated with CS alone or in combination with other products or molecules was assessed;
4. Studies on endosseous implants;
5. Studies that included non-modified animals (osteoporotics, diabetics…).

The exclusion criteria for the study selection were:

1. In vitro studies;
2. Narrative and systematic reviews;
3. Studies that did not use endosseous implants, duplicates, and informatives.

### 2.4. Data Extraction and Analysis

Two independent reviewers (N.L-V., A.L-V.) extracted data from the full texts of the selected articles, including general information, animal parameters (total number, species), chitosan incorporation methods, evaluation moments, analysis methods, conclusions, and implant parameters (total number, length, diameter, shape, location, and characteristics of the implant surface and control) The uncertainty in determining the eligibility of the studies was resolved by discussion between the two reviewers.

### 2.5. Risk of Bias (RoB) of the Selected Articles

SYRCLE’s risk of bias tool (an adapted version of the Cochrane RoB tool with specific biases in animal studies) was used to assess the methodology of the scientific evidence in all the selected studies [21].

### 2.6. Quality of the Reports in the Selected Articles

This assessment involved the modified guidelines provided by Animal Research: Reporting of In Vivo Experiments (ARRIVE) [22], with a total of 23 items. Each item was rated by the reviewers N.L-V. and A.L-V. with scores of 0 (not reported) or 1 (reported), with an overall inventory of all the studies included (Table 1).

## 3. Results

### 3.1. Characteristics of the Studies

**Figure 1:**
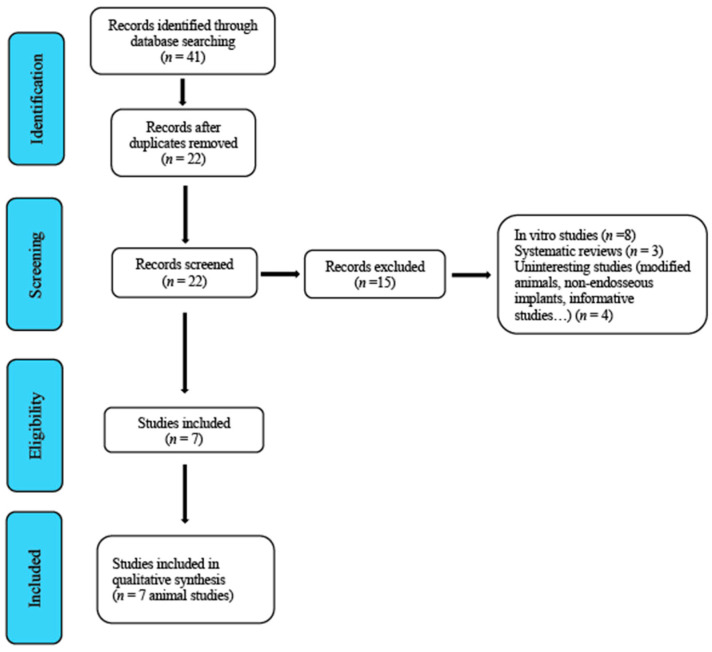
Flowchart.

From 2010 until December 2020, a total of 41 studies were identified and subsequently assessed by the reviewers. After an initial screening, 19 duplicate studies were removed. A second screening led to the removal of 15 studies that were regarded as inadequate because they did not clearly meet the inclusion criteria (Figure 1, Flowchart). Table 2, Table 3 and Table 4 provide a general description of the details of the studies.

### 3.2. Risk of Bias and Quality Assessment of the Animal Studies Included

**Figure 3:**
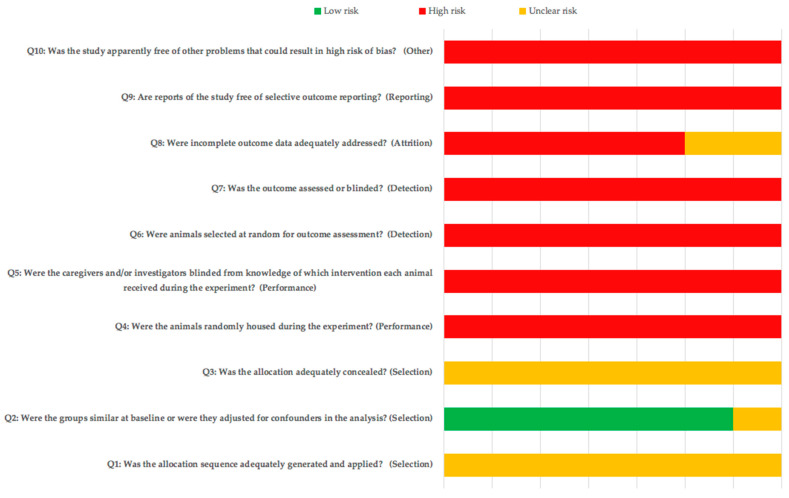
SYRCLE’s (Systematic Review Centre for Laboratory animal Experimentation) risk of bias tool.

The risk of bias assessment results for the animal studies are shown in Figure 3. Although allocation to blinding was mentioned in several articles, the lack of information on the method used resulted in a high and unclear risk of bias for most items. Table 1 shows the ARRIVE guidelines checklist for the animal studies included. The mean score for the studies was 17.14 ± 0.63. All of the studies reported correctly on the title, abstract, introduction, ethical statement, species, surgical procedure, outcomes assessment, and statistical analysis. Items 5 (reasons for animal models), 13 (assignment of animals to experimental groups), 19 (3Rs, Replace, Reduce and Refine.) and 20 (adverse events) were not reported in any of the included studies; only the study by Bhattarai et al. (b) reported limitations in terms of clinical applicability. 

## 4. Discussion

Over time, surface modifications with greater osseoinductive capacity have been developed, with the purpose of overcoming the limitations of traditional Ti surfaces [18].

The most common strategy is the modification of Ti surfaces using biofunctional molecules. This biofunctionalization method involves the deposition of organic and inorganic chemical compounds on the surface with the aim of improving bone-to-implant contact, and thus obtaining an ideal surface capable of full osseointegration capacity and excellent biocompatibility [30,31]. However, certain treatments that are used on Ti surfaces may alter their properties and trigger unknown reactions to a foreign body, affecting the responses of the hard and soft tissues in contact with it; this aspect is largely unknown because of the reduced number of in vivo studies [32].

Although CS is a product that has awakened great interest in the area of biomedical engineering, it has poor solubility in water, which limits its use in living systems [33], where acid solutions such as acetic acid are to be used instead [24,34,35].

All the studies included in our systematic review [23,24,25,26,27,28,29] included an in vitro and an in vivo experimental part using different coatings on Ti surfaces, with CS being among them. 

Marsich and colleagues [28] and Travan and colleagues [29] used lactose-modified chitosan (Chitlac) as a coating for implants, using Ti alloy micro-corrugated implants (Ti6Al4V) as controls in an experimental minipig femur model, reporting the coating’s anti-inflammatory and anti-infective benefits. In this regard, certain authors have recently found evidence of the anti-inflammatory and antioxidant effects of Chitlac in combination with hyaluronic acid on human chondrocytes [36]. Wang and colleagues [23] used a rat experimental model to assess soft tissue healing around CS–collagen-modified Ti surfaces. Soft tissue sealing of the surfaces prevented bacterial invasion, and therefore early dental implant failure [37]. Chen and colleagues [25] assessed the antioxidant and osteogenic capacity of a multilayer surface on Ti substrates (CS–catechol, gelatin, and hydroxyapatite), reporting that multilayered Ti implants were able to promote osteogenesis and osteoblast-related gene expression, and also had remarkable potential to improve the bone–implant interface in vivo. The findings of Georgopoulou and colleagues and Park, Oryan, and colleagues [38,39,40] were consistent with these results, indicating that using a CS–gelatin multicoating on Ti surfaces would increase osteogenic gene expression, providing a promising strategy for bone tissue engineering. Song and colleagues [24] used rat femurs to compare Ti cylinders with others coated with CS, HA (Hyaluronic Acid), and a flavonoid (icariine), reporting higher rates of native bone in the group treated with CS–HA–icariine 2 weeks after implant placement. Certain studies have highlighted the osseoinductive properties of the CS–HA combination due to its favorable bioactive characteristics and mechanical properties to structurally and compositionally reproduce bone tissue [41,42]. Finally, Battarai and colleagues [26,27] conducted two studies based on rat mandibles, both of them assessing Ti coated with gold nanoparticles. The first [26] added a second coating with growth factors and the second [27] with peroxisome proliferator-activated receptor. Both studies reported greater bone formation around the CS-coated implants as compared to the control Ti implants.

Although all the studies included in our review reported favorable results regarding bone growth around Ti implants with one coating of CS [29] or in combination with other coatings [23,24,25,26,27,28], it should be noted that there are a series of limitations concerning the included studies. 

Biomedical researchers use different procedures, which include cell tissue and cultures, experimental animal models, computer simulations, and clinical studies, aimed at mitigating human inconveniences. They all have their advantages and disadvantages, although studies based on animal models have less of the latter than in vitro studies. Some of these shortcomings are the differences in biokinetic parameters or the extrapolation of results to humans; the absence of biokinetics in in vitro methods may lead to misinterpretation of the results [43].

The studies included in our review used both in vivo and in vitro testing; the latter method was not considered because most of the studies involved monoculture research carried out under static growth conditions, bearing no similarity to the conditions of dental implants in humans, which are subject to contact with fluids such as saliva in the oral cavity, making it very difficult to extrapolate the results to the biology of the human body and potentially leading to misleading conclusions. Another significant limitation of in vitro studies is protein concentration in the fluids created in the laboratory. The use of a single host protein or a small selection of them never reflects in vivo oral conditions, which are highly complex [44,45].

Regarding the experimental animal models used in the studies included in our systematic review, 5 studies [23,24,25,26,27] used rodents and two used minipig models [28,29]. In this respect, it should be noted that neither the rodents (rabbits, rats) nor the chosen implant sites (tibia, femur, mandible, etc.) are suitable models to be extrapolated to humans, since cortical remodeling is absent and they stop growing later than other mammals; pigs would perhaps be the most similar animal in terms of bone composition and remodeling [46,47]. 

On the other hand, it would have been desirable to compare experimental coatings with traditional Ti dental implant surfaces (e.g., SLA), with all intraosseous devices being made of Ti of the same purity; some studies used pure Ti [23,24], while others used Ti alloys (Ti6Al4V) [28,29].

Likewise, all the studies included in our review had serious limitations in terms of the number, quality, and methodology of the in vivo studies; precisely, because of the paucity of studies and the complexity of the data they provide, a complementary meta-analysis could not be conducted.

## 5. Conclusions

Bearing in mind the limitations mentioned above, it seems that Ti dental implants coated with CS may have greater osseointegration capacity. It is likely to become a commercial option for the biofunctionalization of dental implants in the future. However, confirmation of this possibility would require well-designed clinical research using broad samples, standardized protocols, and long-term monitoring to support the use of CS as a coating for Ti implants for osteoinduction purposes, and thus to provide surfaces that ensure rapid osseointegration.

## Figures and Tables

**Figure 2:**
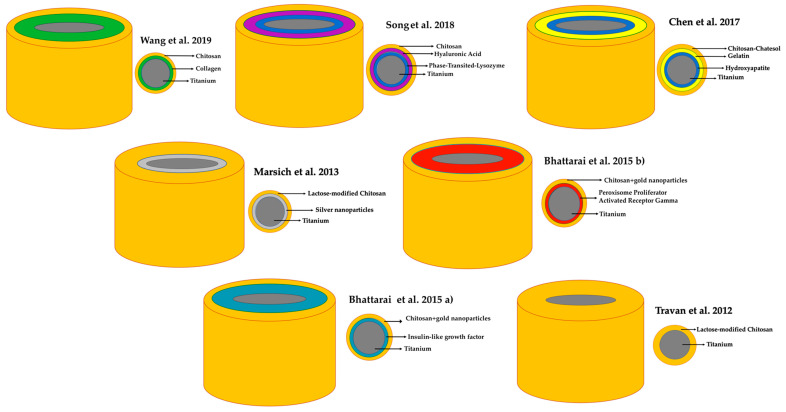
Graphic of CS incorporation to Ti in included studies.

**Table 1:** Checklist of Animal Research: Reporting of In Vivo Experiments (ARRIVE) criteria reported by the included studies.

Studies	Wang et al. 2019 [23]	Song et al. 2018 [24]	Chen et al. 2017 [25]	Bhattarai et al. 2015 (a) [26]	Bhattarai et al. 2015 (b) [27]	Marsich et al. 2013 [28]	Travan et al. 2012 [29]
1. Title	1	1	1	1	1	1	1
**Abstract**							
2. Species	1	1	1	1	1	1	1
3. Key finding	1	1	1	1	1	1	1
**Introduction**							
4. Background	1	1	1	1	1	1	1
5. Reasons for animal models	0	0	0	0	0	0	0
6. Objectives	1	1	1	1	1	1	1
**Methods**							
7. Ethical statement	1	1	1	1	1	1	1
8. Study design	1	1	1	1	1	1	1
9. Experimental procedures	1	1	1	1	1	1	1
10. Experimental animals	1	1	1	1	1	1	1
11. Accommodation and handling of animals	1	0	0	1	1	0	0
12. Sample size	1	1	1	1	1	1	1
13. Assignment of animals to experimental groups	0	0	0	0	0	0	0
14. Anesthesia	1	1	1	1	1	1	1
15. Statistical methods	1	1	1	1	1	1	1
**Results**							
16. Experimental results	1	1	1	1	1	1	1
17. Results and estimation	1	1	1	1	1	1	1
**Discussion**							
18. Interpretation and scientific implications	1	1	1	1	1	1	1
19. 3Rs reported	0	0	0	0	0	0	0
20. Adverse events	0	0	0	0	0	0	0
21. Study limitations	0	0	0	0	1	0	0
22. Generalization/applicability	1	0	1	0	1	1	1
23. Funding	1	1	1	1	0	1	1
TOTAL, SCORE	18	16	17	17	18	17	17

Mode Value: 17.14 ± 0.63. Each item was judged as “0” (not reported) or “1” (reported). The total score for each of the included studies was also recorded.

**Table 2:** Characteristics of included studies.

Studies	Animal Model (n)	Location of Implant Placement	Follow-Up	Analysis Methods	Conclusions
Wang et al. 2019 [23]	Rat model (60)	Mesial (root area of upper right first molar)	4 weeks	- H&E staining. - immunofluorescence staining	The plasmid pLAMA3-CM released from a chitosan/collagen coating was used for adhesion and peri-implant tissue attachment to titanium implants by functioning as a transmucosal barrier.
Song et al. 2018 [24]	Rat model (20)	Femur (midshafts)	2 weeks	- Fluorescence images. - Live/dead staining of cells on different surfaces. - Confocal laser scanning microscopy.	The HA/CS multilayer alone improved surface hydrophilicity. Phase-transited lysozyme nanofilm modulated materials and was applied for surface modification of implants.
Chen et al. 2017 [25]	New Zealand white rabbits (4)	Femora condyles	4 and 12 weeks	- µ-CT analysis - Histochemistry - The percentage of bone-to-implant contact was measured with H&E staining images.	The multilayer coated Ti implants were capable of promoting the proliferation, osteogenesis differentiation, and osteogenesis-related gene expression of osteoblasts and had great potential for clinical implementation in vivo with enhanced osteogenesis at the interface of the bone and implant.
Bhattarai et al. (a) 2015 [26]	Rat model (10)	Mandibles (lower first molar area)	4 weeks	- µ-CT analysis - Immunohistoche- - mistry, hematoxylin and eosin, and tartrate resistance acid phosphatase staining.	The application of CS-GNP/GFBP-3 enhanced bone remodeling around Ti implant surfaces by down-regulating osteoclastogenesis and up-regulating osteogenesis.
Bhattarai et al. (b) 2015 [27]	Rat model (24)	Mandibles (lower first molar area)	1, 2, 3, and 6 weeks	- µ-CT analysis. - Histological evaluation.	Local administration of CS-GNP/PPAR decreases implant-induced inflammation and enhances the expression levels of osteogenic molecules around the implantation site and helps to accelerate bone formation and bone–implant integration.
Marsich et al. 2013 [28]	Minipig model	Femur	8 weeks	- Histological: Sections were cut along the implant axis and stained with the van Gieson method; Olympus BX51TF microscope imaging, Olympus Corp., Tokyo, Japan. - Histomorphometric: The analysis consisted of a quantitative evaluation of the %BIC in the cortical area.	It is assumed that the addition of nAg to the Chitlac coating may have influenced the peri-implant bone response, which was manifested in the absence of lamellar peri-implant bone. The mechanisms are not clear and need further investigation.
Travan et al. [29] 2012	Minipig model	Femur	8 weeks	- Histological: Sections were cut along the implant axis and stained with the van Gieson method; Olympus BX51TF microscope imaging, Olympus Corp., Tokyo, Japan. - Histomorphometric: - A quantitative assessment of the direct (BIC) was performed.	For the Chitlac implants, the total BIC was 72% (min 59%, max 80%). Histomorphometric analysis: Chitlac-TS (nonroughened surface), 72% of the implant interface was in close contact with the cortical bone.

H&E, Hematoxylin&Eosin; Ti, Titanium; GNP, Gold Nanoparticles; GFBP, Growth Factor Binding Protein; PPAR, Peroxisome Proliferator Activated Receptor; BIC, Bone-to-Implant-Contact; nAg, Silver Nanoparticles; Chitlac, lactose derivative of a highly deacetylated chitosan; TS, Unmodified Thermoset; HA, Hyaluronic Acid.

**Table 3:** Characteristics of implants.

Studies	Implants (n)	Implant Dimensions, D(Ø) × L (mm)	Implant Shape	Chitosan Incorporation (See Figure 2)	CS-Modified Implant Surface Characteristics
Wang et al. [23]	16	2 Ø × L 4	Screw	NR	A CS coating was designed to release plasmid DNA and the codeposition of type IV collagen was applied with the purpose of synergistically promoting cellular adhesion and new tissue attachment to the titanium implants.
Song et al. [24]	20	2 Ø × L 2	Ti rods	By immersion in CS solution dissolving 0.1% CS in a 1% acetic acid solution.	Nanofilm coated with multilayer of HA-CS.
Chen et al. [25]	16	3 Ø × L 13	Ti rods	CS solution (3 mg mL^−1^) was prepared with HCl solution (pH 5.0). First, a thin layer of CS was deposited on the Ti surface, followed by three gel–CS bilayers and one HA layer.	Three gel–CS bilayers.
Bhattarai et al. (a) [26]	10	0.85 Ø × 4.5	Screw	For coating with CS-GNP–IGFBP-3 the implants were immersed 10 times in a nanoparticle–DNA solution and frozen at −40 °C.	NR
Bhattarai et al. (b) [27]	24	0.85 Ø × 4.5	Screw	The CS-GNP–PPAR-coated implants were immersed in a nanoparticle–DNA solution and frozen at −240 °C.	NR
Marsich et al. [28]	6	3.6–5 Ø × 8	Truncated cone	Coated with Chitlac or Chitlac–nAg.	NR
Travan et al. [29]		3.6–5 Ø × 8	Truncated cone	Coated with Chitlac or Chitlac–TS.	NR

NR, Not Reported.

**Table 4:** Evaluation of tissues.

Studies, Year	Soft Tissue	Bone Formation
Wang et al. 2019 [23]	Inform through images	Inform through images
Song et al. 2018 [24]	NR	- Fluorescence images of the rat femora after 2 weeks of implant placement. - Bone histology at 2 weeks after implant placement. - Histological analysis of the decalcification samples around Ti.
Chen et al. 2017 [25]	NR	- Bone volume 2 and 4 weeks - Bone-to-implant binding 12 weeks. - New bone formation (area percentage) 2 and 4 weeks. - %BIC2 and 4 weeks.
Bhattarai et al. 2015 (a) [26]	NR	- Bone volume 4 weeks. - Supporting bone around implants.
Bhattarai et al. 2015 (b) [27]	NR	Bone formation around the implant body (inform through images).
Marsich et al. 2013 [28]	NR	BIC for Chitlac–nAg 26% (minimum 22%, maximum 27%)
Travan et al. 2012 [29]	NR	Chitlac-TS implants showed direct bone–implant contact with a minimal soft tissue interlayer, indicating good biological compatibility of the material. For the Chitlac-TS implants, the total BIC was 72% (minimum 59%, maximum 80%)

BIC, Bone-to-Implant Contact; Chitlac-TS, lactose derivative of a highly deacetylated chitosan with unmodified thermoset; Chitlac–nAg, Chitlac–lactose–silver nanoparticles; TS, Thermoset; NR, Not Reported.

## Supplementary Materials

The following are available online at https://www.mdpi.com/2079-7737/10/2/102/s1, Table S1. Database search terms.

## Abbreviations

Ti	Titanium
SLA	Sandblasted, Large-Grit, Acid-Etched
HA	Hyaluronic Acid
CS	Chitosan
CHITLAC	Chitosan–Lactose
BIC	Bone-to-Implant-Contact
H&E	Hematoxylin and Eosin
GNP	Gold Nanoparticles
GFBP	Growth Factor Binding Protein
PPAR	Peroxisome Proliferator-Activated Receptor
nAg	Silver Nanoparticles
TS	Unmodified Thermoset
HCL	Hydrochloric Acid
