# Supplementary material for: Systematic Review of Effectiveness of Chitosan as a Biofunctionalizer of Titanium Implants
Source: Biology (Basel). 2021 Feb 1;10(2):102. doi: 10.3390/biology10020102 (PMC7912802; doi:10.3390/biology10020102)
Supplement: Supplementary file 1 [file biology-10-00102-s001.pdf]

Supplementary materials:

Table S1. Database search terms.

DATABASE	Search Terms
**MEDLINE, CENTRAL, Pudmed**	"titanium"[MeSH Terms] AND "implant"[MeSH Terms] AND "chitosan"[MeSH Terms] AND "coated materials, biocompatible"[MeSH Terms] AND "animals"[MeSH Terms]
**WOS**	titanium implants AND chitosan functionalized surface OR chitosan coating surfaces AND in vivo

MeSH, Medical Subject Headings.
